# Short-Term Outcomes of Revascularization for Chronic Total Occlusions in Multivessel Coronary Artery Disease: Does Coronary Artery Bypass Grafting (CABG) Beat Percutaneous Coronary Intervention (PCI) in Acute Hospital Settings?

**DOI:** 10.7759/cureus.94356

**Published:** 2025-10-11

**Authors:** Loveth Chidiogo Ezefibe, Joshua Nwokoye, Temabore V Daboner, Victor Agommuoh, Udemezue Ezeife, Fidelis E Uwumiro, Oluwatoyin Ayo-Farai, Ihunanya Kanu, Justice C Mgbecheta, Chidera Chukwuneta, Gentle C Uwaoma, Uduakobong Essien

**Affiliations:** 1 Internal Medicine, Nnamdi Azikiwe University College of Health Sciences, Nnewi, NGA; 2 Internal Medicine, Odessa National Medical University, Odessa, UKR; 3 Internal Medicine, Kwame Nkrumah University of Science and Technology, Kumasi, GHA; 4 Internal Medicine, Chukwuemeka Odumegwu Ojukwu University College of Medical Sciences, Anambra, NGA; 5 Internal Medicine, Southern Regional Medical Center, Riverdale, USA; 6 Jiann-Ping Hsu College of Public Health, Georgia Southern University, Statesboro, USA; 7 Internal Medicine, Jackson State University, Jackson, USA; 8 Internal Medicine, Nuvance Health Vassar Brothers Medical Center, Poughkeepsie, USA; 9 Internal Medicine, College of Medicine, University of Nigeria, Enugu, NGA

**Keywords:** acute total occlusion of the left main coronary artery, chronic total occlusion percutaneous coronary intervention (cto pci), complex pci, coronary artery bypass grafting, emergency revascularization with primary percutaneous coronary intervention, high risk cabg, in-hospital adverse outcomes, multivessel coronary artery disease, percutaneous coronary intervention, total coronary occlusion

## Abstract

Introduction: Chronic total occlusions (CTO) are commonly associated with multivessel coronary disease (MVD). Although coronary artery revascularization via percutaneous coronary intervention (PCI) has demonstrated better outcomes than medical therapy alone for single-vessel disease in various clinical trials, the best approach to revascularization in MVD remains complex and less explored. This observational study compared the acute in-hospital outcomes of PCI and coronary artery bypass grafting (CABG) in the revascularization of chronic total occlusion with multivessel disease. Considering the minimally invasive nature of PCI compared to CABG, we hypothesized that PCI would correlate with fewer acute in-hospital complications and a shorter length of hospital stay than CABG for chronic total occlusion in the acute hospital setting.

Methods: We analyzed 151,270 hospitalizations for CTO with MVD across the U.S from the 2017 to 2022 national inpatient sample registry. Approximately 105,925 (70%) hospitalizations underwent PCI and 45,345 (30%) underwent CABG. Propensity score matching was used to alleviate the residual bias from non-randomized treatment assignments. Mortality and complication rates were compared using the McNemar’s test. Hospital stay duration and total costs were compared using paired sample t-tests or Wilcoxon rank-sum tests.

Results: The study cohort consisted of 101,141 men (66.2%) and 104,830 White Americans (69.3%), with a median age of 74 years (interquartile range [IQR], 65-82). All hospitalizations in this cohort were associated with an estimated 10-year mortality rate of 25% or less (Charlson Comorbidity Index [CCI] score ≥3). The matched cohort was comprised of 9,210 CABGs and 9,210 PCIs. All covariate imbalances were alleviated. Compared to PCI, CABG was associated with higher mortality (442, 4.8% vs. 313, 33.4%; P=0.003), longer hospital stay (15 vs. 5 days; P<0.001), and higher hospitalization costs (mean: $320,917 ±$24,158 vs. $129,396 ± $7,234; P<0.001). CABG also showed higher postprocedural incidences of acute ischemic stroke (157, 1.7% vs. 111, 1.2%; P=0.008), complete atrioventricular block (147, 1.6% vs. 111, 1.2%; P=0.035), sepsis (332, 3.6% vs. 166, 1.8%; P<0.001), atrial flutter (1,649 [17.9%] vs. 635 [6.9%]; P<0.001), atrial fibrillation (3,039 [33%] vs. 1,382 [15%]; P<0.001), and ventricular fibrillation (193 [2.1%] vs. 129 [1.4%]; P=0.046). However, repeat acute myocardial infarction rates were lower with CABG than with PCI (2.7% vs. 4.3%; P<0.001).

Conclusion: PCI was associated with lower in-hospital mortality, shorter duration of hospitalization, and decreased hospitalization costs compared to CABG. Conversely, CABG was linked to lower rates of recurrent myocardial infarction but exhibited higher incidences of acute postprocedural arrhythmias, stroke, and sepsis.

## Introduction

Chronic total occlusion with multivessel disease (CTO-MVD) is defined by a complete cessation of antegrade coronary flow in multiple vessels persisting for more than three months, as confirmed through angiographic evidence or clinical suspicion. This condition constitutes a distinct category of coronary artery disease (CAD), identified in approximately 10-30% of diagnostic coronary angiograms, and poses considerable challenges for coronary interventions [1,2]. Patients with CAD, including those with CTOs, are at an increased risk of mortality and cardiovascular events, particularly when other comorbidities are present. These risks are further exacerbated following revascularization procedures, such as percutaneous coronary intervention (PCI) or coronary artery bypass grafting (CABG) [3-6]. Additionally, MVD is frequently observed in patients with CTOs, with previous studies indicating prevalence rates ranging from 51.8% to 86.5% [7-12]. While PCI is the standard treatment for single-vessel disease, selecting the optimal revascularization strategy in MVD is more complex, and the outcomes are less extensively studied.

While previous research has predominantly concentrated on long-term outcomes such as survival and recurrent ischemic events, acute in-hospital outcomes are particularly pertinent for this population due to their comorbidity burden, and increased peri-procedural risks. For many patients, particularly those with limited life expectancy or significant frailty, short-term safety, procedural complications, hospital length of stay, and associated costs may hold greater clinical significance than mid- or long-term endpoints.

The presence of multivessel disease significantly complicates both PCI and CABG in patients with CTO. In this context, PCI is frequently complicated by technical challenges, such as extended lesion lengths, substantial calcification, and difficulties in achieving complete revascularization across multiple occluded territories. These factors collectively contribute to reduced procedural success and increased rates of restenosis. PCI in this setting may result in incomplete revascularization, especially in diffuse, calcified, or complex lesions, increasing the risk of myocardial infarction and repeat procedures. Conversely, CABG in patients with MVD and CTO entails increased operative risk due to the necessity for multiple grafts, prolonged bypass times, and an increased likelihood of perioperative complications, particularly in individuals of advanced age or with comorbid conditions. CABG can bypass multiple lesions but carries higher surgical complexity and perioperative stroke risk, particularly in patients with left main disease, chronic total occlusions, or significant comorbidities such as diabetes, reduced ventricular function, or advanced age [13-15]. These complexities create a unique clinical dynamic characterized by uncertainty in selecting the optimal revascularization strategy. There is a need for comparative evidence to inform decision-making in this high-risk population.

This observational study evaluates the acute in-hospital outcomes of PCI versus CABG in patients with CTO and MVD. The aim is to clarify the relative benefits of each strategy, providing evidence to guide treatment decisions in situations where short-term hospitalization risks may outweigh the potential long-term advantages of surgical durability. Given PCI’s less invasive nature compared to CABG, we hypothesized that PCI would be associated with fewer acute complications and a shorter length of stay during hospitalization. The primary endpoints of interest were mortality rate, length of hospital stay and total hospital costs. Secondary endpoints include acute kidney injury, repeat myocardial infarction, acute stroke, heart block, sepsis, and new onset cardiac arrhythmias.

## Materials and methods

Study data and selection criteria

Data on hospitalizations involving CABG and PCI procedures performed across the U.S were collected from the Nationwide Inpatient Sample (NIS) database. The NIS is the largest publicly available inpatient data repository in the United States. It captures data on approximately 35 million annual hospitalizations across the United States and records data on the location, teaching status, and hospital size. It provides extensive data on healthcare utilization and outcomes, including the duration of hospital stay, total charges, complications, and patient outcomes. The NIS records up to 40 different diagnoses and 15 procedures for each hospital stay using the International Classification of Diseases, Tenth Revision, Clinical Modification, and Procedure Coding System (ICD-10-CM/PCS), ensuring that each case was anonymized and recorded as a distinct entry. It records information on patient demographics, comorbidities, procedures, insurance status, total hospital charges, and in-hospital outcomes, allowing comprehensive multiyear analyses [16-19].

The United States transitioned to ICD-10-CM/PCS on October 1, 2015. Accordingly, we used only the ICD-10-CM codes for the index study to ensure consistent coding conventions. The 2017-2022 NIS database was thus queried for all adult hospitalizations with a primary diagnosis of CTO with multivessel disease (CTOMVD) using relevant ICD-10 diagnostic codes sourced from official WHO websites (Appendix 1). Hospitalizations associated with PCI or CABG were subsequently identified using ICD-10 procedure codes. The total cohort was subsequently divided into two groups for comparison: hospitalizations with PCI and those with CABG. Hospitalizations without records of either the procedure or incomplete data were excluded. To reduce inclusion of readmissions in the study cohort, all hospitalizations with previous PCI or CABG were excluded (Appendix 1). Our coding approach is in line with the methodologies established in prior research [20]. CTO was defined using ICD-10 code I25.82. For code validation, two independent authors collated all relevant ICD-10-CM/PCS codes, achieving a discrepancy rate of less than 2%. A third author reviewed and refined the codes exhibiting discrepancy by comparing them with validated codes from previously published studies and making iterations as needed to ensure accuracy. To ensure adherence to acceptable conventions in our study, we adopted the study design checklist for research based on the NIS, as proposed by Khera et al. in 2017 (Appendix 3) [21].

**Figure 1:**
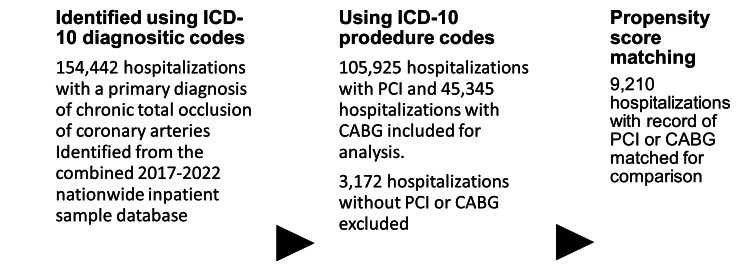
Study sample selection ICD-10, International Classification of Diseases, Tenth Revision; CTO, chronic total occlusion; PCI, percutaneous coronary syndrome; CABG, coronary artery bypass grafting

Figure 1 summarizes the study selection process.

This study did not require Institutional Review Board (IRB) approval as it utilized publicly available, de-identified NIS data, which had already received ethical clearance from the Agency for Healthcare Research and Quality. The anonymization of patient and hospital data within the repository obviates the requirement for obtaining individual patient consent. All pertinent data supporting this study are included in the manuscript.

Outcomes

The primary outcomes of interest were all-cause in-hospital mortality, total hospital cost (THC), and length of hospital stay (LOS). Mortality was recorded as a dichotomous variable within the NIS, whereas LOS and THC (recorded in US dollars) were recorded as continuous variables. Secondary outcomes included acute in-hospital complications, such as acute myocardial infarction, acute ischemic stroke, complete atrioventricular block, septicemia, atrial arrhythmias, and ventricular fibrillation, all of which were identified using ICD-10 and ICD-9 diagnostic codes as appropriate.

Statistical analysis

This study was designed to compare the outcomes of PCI with those of CABG in terms of mortality, LOS, hospital costs, and in-hospital complications. The unit of analysis was the individual hospitalization, consistent with the structure of the NIS database, which records each admission as a distinct entry. To account for clustering within hospitals and potential correlations among patients treated at the same institution, we applied survey-weighted analyses using the NIS-provided hospital clustering variables. Clustering was addressed by using the svyset command with the hospital identifier as the clustering variable to account for intra-hospital correlations among patients. Patient-level discharge weights were then applied via the pweight option to produce nationally representative estimates. Additionally, stratification variables, including hospital type and location, were incorporated into the svyset command to ensure precise variance estimation. Continuous variables are expressed as mean (standard deviation [SD]) or median (interquartile range [IQR]), while categorical variables are presented as absolute numbers and percentages. For univariate comparisons, Pearson’s χ2 analysis was employed to evaluate categorical variables; conversely, continuous variables were analyzed using the independent samples t-test and Wilcoxon rank sum test for normally and non-normally distributed data, respectively. The normality of the numerical variables was assessed using the Kolmogorov-Smirnov test, graphical tests, and descriptive statistics. A two-stage analysis was performed using the unmatched and propensity score-matched cohorts. In the primary analysis (unmatched model), stepwise univariate logistic regression analyses identified covariates associated with the primary and secondary outcomes of interest (P≤0.05 for entry; P>0.10 for removal). Subsequent multivariable logistic regression analysis was conducted, accounting only for factors significantly associated with the outcomes of interest, including the following covariates: age, sex, Charlson Comorbidity Index (CCI), illness severity, race, insurance status, hospital region, type of admission, and comorbidities. Hospital costs were adjusted to reflect the 2022 US dollar using the Medical Expenditure Panel Survey Index. 

We evaluated the burden of comorbidities utilizing the open-source CCI score, incorporating it with variables such as age, sex, and the requirement for mechanical ventilation to account for illness severity. The predictive capacity of this method for mortality is comparable to that of the Acute Physiology and Chronic Health Evaluation (APACHE) II score [22]. Specifically, we employed Sundararajan’s adaptation of the modified Deyo’s CCI, which classifies comorbidities into four categories based on increasing mortality risk, rendering it suitable for population-based studies. A CCI score exceeding 3 signifies a 25% 10-year mortality rate, while scores of 2 and 1 correspond to 10% and 4% 10-year mortality rates, respectively [23,24]. Furthermore, we assessed illness severity and baseline mortality risk using the All Patient Refined-Diagnosis Related Groups (APR-DRG) severity of illness and risk of mortality classifications available as unique variables within the NIS database. In research utilizing administrative databases, these APR-DRG categories have been demonstrated to be effective indicators of illness severity and predictors of in-hospital mortality. They provide a robust complement to traditional indices such as the CCI and the Elixhauser comorbidity index [25,26].

Collinearity was evaluated by calculating the variance inflation factor for each independent variable, with values exceeding 5 indicating collinearity. Variables exhibiting collinearity were excluded to enhance the stability of the logistic regression model. Propensity score matching is employed to mitigate treatment selection bias when estimating causal treatment effects in non-randomized studies [27]. The CABG and PCI cohorts were matched based on a set of variables that could otherwise confound their comparisons. Once a matched sample is established, the treatment effect can be estimated by directly comparing the primary and secondary outcomes between the two groups within the matched sample [28]. In our secondary analysis (matched model), propensity scores were computed by accounting for all factors that were significantly different between the PCI and CABG cohorts in the unmatched model or significantly associated with undergoing CABG in the logistic regression analysis. Consequently, individual propensity scores were calculated using logistic regression modeling [29] based on the following covariates: age, sex, race, insurance type, hospital location, teaching status, illness severity, mortality risk, geographic region, and comorbidities. The PCI and CABG cohorts were then paired 1:1 on these propensity scores using exact matching [30]. A standard caliper size of 0.2 × log [SD of the propensity score] was utilized. Standardized differences were estimated before and after matching to assess the balance of covariates; small absolute values (<0.1) indicated a balance between treatment groups.

**Figure 2:**
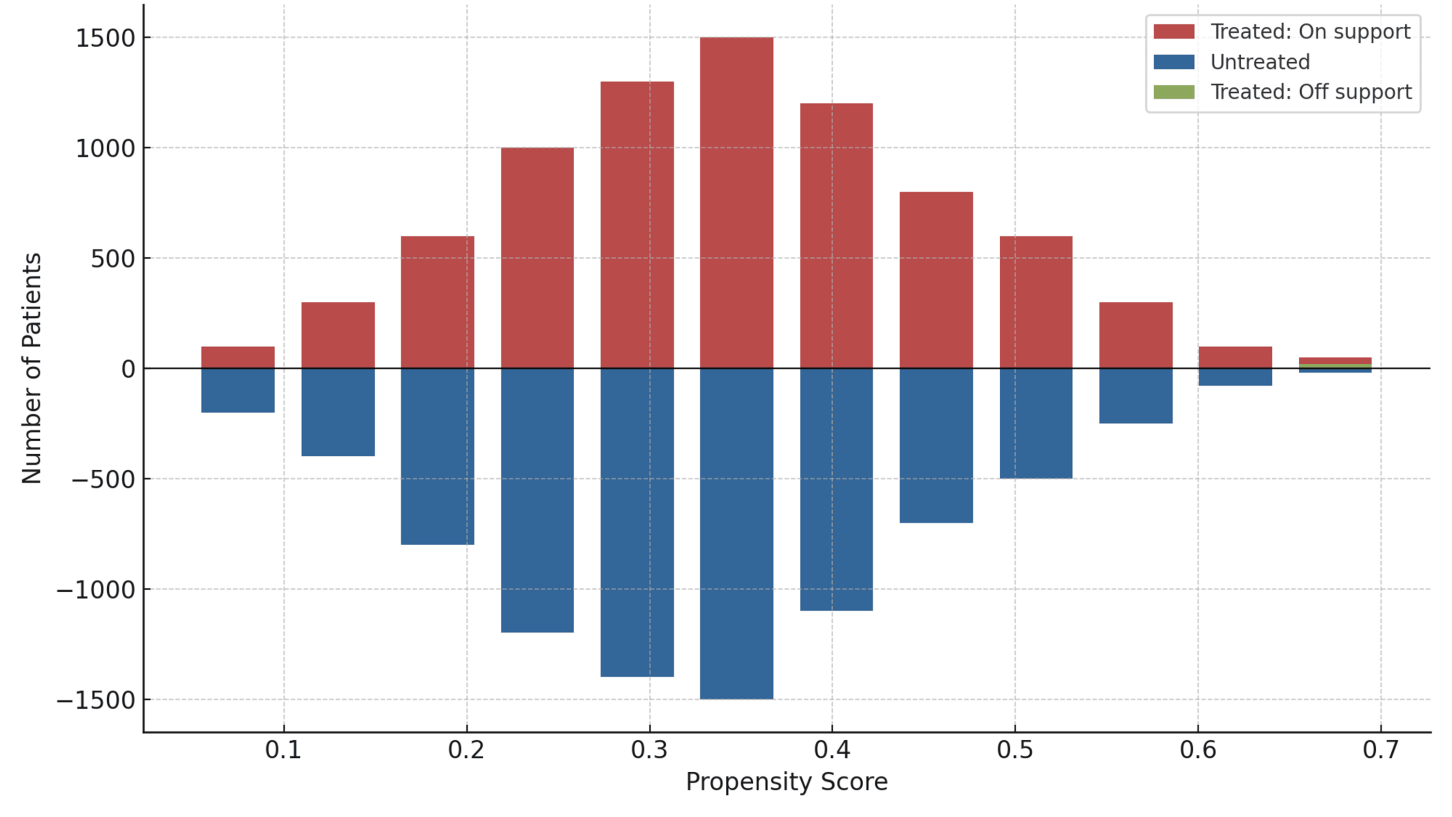
Graphical test of covariate balance after propensity score matching This image is a graphical diagnostic of balance following propensity score matching, displaying the distribution of propensity scores for coronary artery bypass grafting (CABG) and percutaneous coronary intervention (PCI) individuals. The x-axis represents the estimated propensity scores, while the y-axis indicates frequency. Red bars above the horizontal axis represent treated individuals who were successfully matched (on support), blue bars below the axis represent untreated individuals, and the small green bar denotes treated individuals who had no suitable untreated match and were excluded from analysis (off support). The overlap between the treated and untreated groups, especially in the 0.15 to 0.55 range, indicates adequate common support, which is essential for unbiased treatment effect estimation. The absence of large unmatched regions suggests good balance and that most comparisons between groups are valid. This plot confirms that matching was successful for the majority of treated individuals, with only a small portion outside the region of comparability.

The post-match balance of the covariates was evaluated both graphically and statistically (Figure 2, Appendix 4). Following 1:1 propensity score matching, the incidence of mortality and complications between matched PCI and CABG patients was analyzed using conditional logistic regression. Continuous outcomes, such as the length of hospital stay and total hospital costs, were compared between the matched PCI and CABG cohorts using a paired sample t-test. Statistical significance was determined at P ≤ 0.05, with all tests being two-sided. All statistical analyses were conducted using Stata statistical software (version 7.0MP; Stata Corp LLC, College Station, TX, USA), with propensity score matching executed via the Stata psmatch2 extension program developed by Edwin Leuven and Barbara Sianesi [31].

A sensitivity analysis was deemed unnecessary in this study for several reasons. Firstly, the dataset was derived from a large, nationally representative sample with a high event count, thereby minimizing the influence of outliers or small-study effects that sensitivity analyses typically address. Secondly, rigorous propensity score matching was employed to balance baseline characteristics and eliminate covariate imbalances between the PCI and CABG cohorts, effectively controlling for key sources of heterogeneity. Thirdly, the inclusion criteria were narrowly defined (patients with CTO and multivessel disease), ensuring a clinically homogeneous population and reducing variability that would otherwise necessitate robustness testing. Finally, all analyses were conducted on a matched cohort, where repeating sensitivity analyses (such as leave-one-out or subgroup exclusions) would provide little additional insight beyond the balanced comparison already achieved.

Missing data

Missing data variables in the study cohort were identified using the "mdesc" Stata command, with a threshold of less than 5% deemed acceptable a priori (Appendix 2). We elected to exclude all hospitalizations with missing data if the overall level of data missingness is >5%. Excluding this small fraction of records reduces computational complexity and avoids the assumptions associated with imputation methods, such as overfitting, underestimation of variability, biased correlations, and inconsistent sample size estimations. The proportion of missing data in the index study was found to be < 1%, obviating the need for imputation or adjustment of missing data. Therefore we included and analyzed all hospitalizations initially included in the study cohort. When missingness affects less than 5% of the study population, it is generally considered negligible, as it exerts minimal impact on effect estimates, statistical power, and overall validity [32,33]. 

## Results

The mean level of covariate missingness among variables was 0.55% (832 variables), with "race" exhibiting the highest proportion of missing data at 2.91% (4,401 hospitalizations). A complete analysis of the study cohort was conducted due to the minimal proportion of missing covariates (less than the predefined threshold of <5%).

We analyzed 151,270 hospitalizations for MVD CTO who underwent PCI or CABG. Approximately 45,345 hospitalizations involved CABG, and 105,925 patients underwent PCI. The overall cohort consisted of 100,141 (66.2%) males, 104,830 (69.3%) White Americans, and 20,875 (13.8%) Black Americans, with a median age of 74 years (IQR: 65-82 years). All patients in the study cohort had a CCI of ≥3. Table 1 summarizes the demographic characteristics of the patient cohorts undergoing PCI and CABG before propensity score matching.

**Table 1:** Patient- and Hospital-level Demographics of PCI and CABG Patients Before Propensity Score Matching The data presents demographic and hospital level variables before and after propensity score matching to demonstrate alleviation of baseline confounding. Variables in the PCI and CABG cohorts are presented as frequencies (n) and percentages (%) for categorical variables and median with interquartile range (IQR) for continuous variables. Values are presented as N (%) unless indicated otherwise HF, heart failure; CCI, Charlson comorbidity index; MI, myocardial infarction; COPD, chronic obstructive pulmonary disease; CCF, congestive cardiac failure; CABG, coronary artery bypass grafting; PCI, percutaneous coronary intervention; MI, myocardial infarction; HMO, health maintenance organization; LOD, likelihood of dying; LOF, loss of function; DRG, diagnosis-related groups. Chi-square tests were used to compare categorical sociodemographic variables and t-tests were used to compare continuous variables P-values are considered significant at p<0.01

Variables	PCI (N=105,925) n (%) Unless Otherwise Specified	CABG (N=45,345) n (%) Unless Otherwise Specified	P-value
Median age (years)	68.6	70.8	<0.001
Sex			<0.001
Male	67,368 (63.6)	32,830 (72.4)	-
Female	38,557 (36.4)	12,515 (27.6)	-
Insurance type			<0.001
Medicare	82,304 (77.7)	32,467 (71.6)	-
Medicaid	6,779 (6.4)	3,537 (7.8)	-
Private, including HMO	15,147 (14.3)	8,298 (18.3)	-
Self-pay	1,695 (1.6)	1,043 (2.3)	-
Race			<0.001
White	73,512 (69.4)	31,243 (68.9)	-
Black	15,465 (14.6)	5,396 (11.9)	-
Hispanic	9,745 (9.2)	4,897 (10.8)	-
Asian/Pacific Islanders	3,284 (3.1)	2,131 (4.7)	-
Native Americans	847 (0.8)	272 (0.6)	-
others	3,072 (2.9)	1,451 (3.2)	-
Nonelective admissions	100,841 (95.2)	41,491 (91.5)	<0.001
Weekend admissions	26,693 (25.2)	10,429 (23.0)	<0.001
All Patient Refined- DRG: Severity of illness			<0.001
Minor LOF	4,873 (4.6)	91 (0.2)	-
Moderate LOF	35,485 (33.5)	6,575 (14.5)	-
Major LOF	45,018 (42.5)	21,856 (48.2)	-
Extreme LOF	20,550 (19.4)	16,778 (37.0)	-
All Patient Refined- DRG: Risk of Mortality			<0.001
Minor LOD	7,203 (6.8)	45 (0.1)	-
Moderate LOD	35,273 (33.3)	8,117 (17.9)	-
Major LOD	41,840 (39.5)	19,634 (43.3)	-
Extreme LOF	21,503 (20.3)	17,549 (38.7)	-
Hospital region			<0.001
Northeast	15,783 (14.9)	7,391 (16.3)	-
Midwest	27,223 (25.7)	9,749 (21.5)	-
South	43,111 (40.7)	18,954 (41.8)	-
West	19,914 (18.8)	9,250 (20.4)	-
Admission to urban teaching hospitals	77,643 (73.3)	36,865 (81.3)	<0.001
Hospital control/ownership			0.059
government, nonfederal	8,156 (7.7)	3,129 (6.9)	-
private, nonprofit	80,821 (76.3)	35,278 (77.8)	-
private, investor-owned	16,948 (16.0)	6,938 (15.3)	-
Obesity	14,724 (13.9)	7,981 (17.6)	<0.001
Hypertension	1,843 (1.74)	775 (1.71)	0.890
Dyslipidemia	69,063 (65.2)	29,565 (65.2)	0.906
Tobacco use	46,395 (43.8)	19,725 (43.5)	0.652
Peripheral vascular disease	21,397 (20.2)	8,525 (18.8)	0.007
CCF	63,343 (59.8)	28,386 (62.6)	<0.001
Previous Stroke	8,474 (8.0)	6,983 (15.4)	<0.001
COPD	26,799 (25.3)	11,699 (25.8)	0.358
Dementia	2,013 (1.9)	1,950 (4.3)	<0.001
Types 1 and 2 diabetes	68,216 (64.4)	30,290 (66.8)	<0.001
Chronic liver disease	2,966 (2.8)	1,270 (2.8)	0.990
Malignant neoplasia	3,707 (3.5)	1,270 (2.8)	0.004
Rheumatoid disease	2,648 (2.5)	1,315 (2.9)	0.054
Hemiplegia	741 (0.7)	589 (1.3)	<0.001

Table 2 summarizes the baseline demographic characteristics of the patient cohorts undergoing PCI and CABG after propensity score matching.

**Table 2:** Patient- and Hospital-level Demographics of PCI and CABG Patients After Propensity Score Matching The data presents demographic and hospital level variables before and after propensity score matching to demonstrate alleviation of baseline confounding. Standard error <0.5 was considered minimum acceptable variability in post-match cohort. Variables in the PCI and CABG cohorts are presented as frequencies (n) and percentages (%) for categorical variables and median with interquartile range (IQR) for continuous variables. Values are presented as N (%) unless indicated otherwise HF, heart failure; CCI, Charlson comorbidity index; MI, myocardial infarction; COPD, chronic obstructive pulmonary disease; CCF, congestive cardiac failure; CABG, coronary artery bypass grafting; PCI, percutaneous coronary intervention; MI, myocardial infarction; HMO, health maintenance organization; LOD, likelihood of dying; LOF, loss of function; DRG, diagnosis-related groups. Chi-square tests were used to compare categorical sociodemographic variables and t-tests were used to compare continuous variables P-values are considered significant at p<0.01

Variables	PCI (N=9,210) n (%) Unless Otherwise Specified	CABG (N=9,210) n (%) Unless Otherwise Specified	P-value	χ2	Standard Error
Median age (years)	66.0	65.9	0.897	5.00	0.08
Sex			0.999	0.00	0.07
Male	5,646 (61.3)	5,646 (61.3)	-		-
Female	3,564 (38.7)	3,564 (38.7)	-		-
Insurance type	Insurance type		0.852	0.309	0.04
Medicare	5,526 (60.0)	5,489 (59.6)	-		-
Medicaid	1,133 (12.3)	1,124 (12.2)	-		-
Private, including HMO	2,183 (23.7)	2,192 (23.8)	-		-
Self-pay	378 (4.1)	405 (4.4)	-		-
Race	Race		0.979	0.02	0.01
White	4,623 (50.2)	4,660 (50.6)	-		-
Black	1,953 (21.2)	1,906 (20.7)	-		-
Hispanic	1,363 (14.8)	1,335 (14.5)	-		-
Asian/Pacific Islanders	571 (6.2)	571 (6.2)	-		-
Native Americans	166 (1.8)	120 (1.3)	-		-
others	534 (5.8)	525 (5.7)	-		-
Nonelective admissions	8,335 (90.5)	8,289 (90.0)	0.981	0.02	0.006
Weekend admissions	6,346 (68.9)	6,198 (67.3)	0.880	0.03	0.08
All Patient Refined- DRG: Severity of illness	All Patient Refined- DRG: Severity of illness		0.942	0.04	0.03
Minor LOF	433 (4.7)	414 (4.5)	-		-
Moderate LOF	3,131 (34.0)	3,030 (32.9)	-		-
Major LOF	3,896 (42.3)	3,850 (41.8)	-		-
Extreme LOF	1,750 (19.0)	1,916 (20.8)	-		-
All Patient Refined- DRG: Risk of Mortality	All Patient Refined- DRG: Risk of Mortality		0.868	0.05	0.07
Minor LOD	599 (6.5)	599 (6.5)	-		-
Moderate LOD	3,352 (36.4)	3,352 (36.4)	-		-
Major LOD	3,472 (37.7)	3,095 (33.6)	-		-
Extreme LOF	1,778 (19.3)	2,164 (23.5)	-		-
Hospital region	Hospital region		>0.999	0.02	0.01
Northeast	1,207 (13.1)	1,253 (13.6)	-		-
Midwest	1,851 (20.1)	1,759 (19.1)	-		-
South	4,108 (44.6)	4,052 (44.0)	-		-
West	2,045 (22.2)	2,146 (23.3)	-		-
Admission to urban teaching hospitals	6,429 (69.8)	6,254 (67.9)	0.878	0.07	0.02
Hospital control/ownership	Hospital control/ownership		0.910	0.03	0.10
government, nonfederal	847 (9.2)	700 (7.6)	-		-
private, nonprofit	6,788 (73.7)	6,880 (74.7)	-		-
private, investor-owned	1,667 (18.1)	1,630 (17.7)	-		-
Comorbidities					
Obesity	2,210 (24.0)	2,220 (24.1)	0.868	0.03	0.01
Hypertension	147 (1.6)	166 (1.8)	>0.999	0.01	0.03
Dyslipidemia	6,005 (65.2)	6,023 (65.4)	0.882	0.03	0.05
Tobacco use	4,034 (43.8)	3,896 (42.3)	0.886	0.03	0.002
Peripheral vascular disease	1,630 (17.7)	1,547 (16.8)	>0.999	0.02	0.04
CCF	5,323 (57.8)	5,222 (56.7)	>0.999	0.02	0.03
Previous Stroke	810 (8.8)	1,326 (14.4)	0.375	0.03	0.08
COPD	2,238 (24.3)	2,422 (26.3)	0.870	0.03	0.009
Dementia	276 (3.0)	120 (1.3)	0.757	0.04	0.04
Types 1 and 2 diabetes	6,097 (66.2)	6,005 (65.2)	>0.999	0.02	0.10
Chronic liver disease	295 (3.2)	322 (3.5)	>0.999	0.02	0.01
Malignant neoplasia	267 (2.9)	221 (2.4)	>0.999	0.02	0.03
Rheumatoid disease	276 (3.0)	240 (2.6)	0.746	0.04	0.09
Hemiplegia	92 (1.0)	111 (1.2)	0.745	0.04	0.04

Factors predicting CABG versus PCI

Prior to implementing propensity score matching, both patient- and hospital-level variables were incorporated into a multivariable logistic regression model to ascertain the predictors of CABG. The analysis identified several variables that were independently associated with the likelihood of undergoing CABG: Asian/Pacific Islander ethnicity (odds ratio [OR], 1.28 [95% CI, 1.10-1.48]; P<0.001), type of insurance (Private, including HMO, OR, 1.27 [95% CI, 1.17-1.38]; P<0.001; Self-pay, OR, 1.39 [95% CI, 1.13-1.71]; P<0.001), presence of dyslipidemia (OR, 1.10 [95% CI, 1.04-1.17]; P=0.001), obesity (OR, 1.36 [95% CI, 1.04-1.17]; P=0.001), elective admission status (OR, 1.92 [95% CI, 1.04-1.17]; P=0.001), treatment at an urban teaching hospital (OR, 1.43 [95% CI, 1.32-1.54]), cerebrovascular disease (OR, 1.84 [95% CI, 1.68-2.02]), and the severity of illness (moderate loss of function, OR, 7.49 [95% CI, 3.70-9.36]; P<0.001; major loss of function, OR, 8.57 [95% CI, 6.15-18.80]; P<0.001; extreme loss of function, OR, 38.8 [95% CI, 33.55-70.62]; P<0.001).

Propensity score-matched cohort

We employed a 1:1 matching strategy to compare PCI and CABG, predicated on the identified predictors for a higher propensity to undergo CABG and variables exhibiting significant differences between the two groups in the initial unmatched cohorts (P-value < 0.05, as detailed in Table 1). Consequently, the propensity score-matched cohort in our primary analysis comprised 18,420 patients, evenly divided between the PCI and CABG groups, each containing 9,210 (50%) patients. Following the matching process, disparities in patient- and hospital-level covariates, including age, sex, race, primary payer, hospital region, teaching status, timing of hospitalization (weekend vs. weekday), admission type (elective vs. non-elective), disease severity, mortality risk, and comorbidities, were significantly mitigated between the PCI and CABG cohorts. The effectiveness of the matching procedure was evident as it reduced the absolute standardized differences for all covariates to below 10%, achieving a median bias reduction of 0.03 (Table 1). Figure 2 illustrates the balance of the matched covariates between the CABG and PCI groups.

Impact of PCI versus CABG on primary outcomes in a propensity score-matched cohort

In the propensity score-matched cohort, the mean duration of hospitalization was 9.8 days (standard deviation [SD] 3.6), with a total of 875 mortalities (4.8%). Specifically, the CABG cohort experienced approximately 535 mortalities (5.8%), whereas the PCI cohort had 340 mortalities (3.4%; P=0.003). Consistent with the results from the unmatched cohort of 151,270 hospitalizations (Table 3), the CABG cohort was associated with a significantly higher mortality rate (P=0.003; Table 4).

**Table 3:** PCI versus CABG on primary and secondary outcomes: unmatched analysis Variables in the table are presented as N (%) for categorical variables and mean +/- standard deviation for continuous variables †Adjusted for sex, elective vs. nonelective admission, age, race, primary payer, hospital region, comorbidities, illness severity, risk of mortality, and Charlson comorbidity index. P-values are significant at values <0.05 The z-score’s sign shows the direction of effect (positive or negative). Values with z > 1.96 are statistically significant at p < 0.05. Very high z-values (e.g. >10) suggest very strong evidence against the null hypothesis * Adjusted mean difference aOR, adjusted odds ratio; CI, confidence interval; LOS, length of hospital stay; THC, total hospital costs; US$, United States dollars; CABG, coronary artery bypass grafting; PCI, percutaneous coronary intervention; MI, myocardial infarction; MI, myocardial infarction; AV, atrioventricular adjusted odds ratios are calculated using multivariable logistic regression analysis

Outcomes	PCI (N=105,925), n (%)	CABG (N=45,345), n (%)	aOR (95% CI)^ †^	z-score	P-value
Primary outcomes					
Inpatient mortality, n (%)	3,574 (3.4)	2,165 (4.8)	0.81 (0.70-0.93)	2.91	0.003
LOS, days, mean	5.3	14.4	9.8* (8.4-11.2)	-	<0.0001
THC, US$, mean (SD)	123,996 (116,583)	303,013 (248,785)	227,945* (195,614-260,276)	-	<0.0001
Secondary outcomes					
Acute kidney injury	37,280 (35.2)	20,345 (44.9%)	1.03 (0.94-1.06)	0.96	0.992
Postoperative dialysis	8,615 (8.1)	4,110 (9.1)	1.23 (1.11-1.37)	3.05	<0.001
Acute MI	630 (0.6)	190 (0.4)	0.64 (0.41-0.99)	-1.90	0.665
Acute ischemic stroke	825 (0.8)	680 (1.5)	0.78 (0.58-1.04)	-1.65	0.089
Complete AV block	1,410 (1.3)	905 (2.0)	0.79 (0.30-1.86)	-0.50	0.589
Sepsis	1,915 (1.8)	1,625 (3.6)	0.90 (0.72-1.11)	-0.95	0.319
Atrial flutter	9,345 (8.8)	9,079 (20)	2.36 (2.17-2.56)	20.42	<0.001
Atrial fibrillation	20,120 (19.0)	16,135 (35.6)	2.28 (2.14-2.43)	25.72	<0.0001
Ventricular fibrillation	1,670 (1.6)	1,000 (2.2)	0.84 (0.68-1.03)	-1.67	0.119

**Table 4:** Comparison of Clinical Outcomes between PCI and CABG: Matched Cohort of 18,420 Hospitalizations PCI, percutaneous coronary intervention; CABG, coronary artery bypass grafting; THC, total hospital costs; LOS, length of stay; US, United States; MI, myocardial infarction; AV, atrioventricular Variables in the table are presented as N, (%) for categorical variables and mean +/- standard deviation for continuous variables adjusted odds ratios are calculated using multivariable logistic regression analysis p values are considered significant at p<0.05 ** t-score

Outcomes	PCI (N=9,210), n (%)	CABG (N=9,210), n (%)	z-score	P-value
Primary outcomes				
Inpatient mortality, n (%)	340 (3.4)	535 (5.8)	-3.00	0.003
LOS, days, mean	5.9	15.0	-77.1**	<0.0001
THC, US$, mean (SD)	129,396 (6,458)	320,917 (75,696)	-234.1**	<0.0001
Secondary outcomes				
Acute kidney injury	2,720 (29.5)	2,940 (31.9)	-2.54	0.011
Post-operative dialysis	910 (9.9)	695 (7.5)	4.24	<0.001
Acute MI	400 (4.3)	250 (2.7)	5.13	<0.0001
Acute stroke	115 (1.2)	160 (1.7)	-2.67	0.008
Complete AV block	110 (1.2)	145 (1.6)	-2.10	0.035
Sepsis	170 (1.8)	330 (3.6)	-4.08	<0.001
Atrial flutter	635 (6.9)	1,645 (17.9)	-18.15	<0.001
Atrial fibrillation	1,385 (15.0)	3,035 (33.0)	-21.29	<0.0001
Ventricular fibrillation	135 (1.5)	190 (2.1)	-2.34	0.019
PCI, percutaneous coronary intervention; CABG, coronary artery bypass grafting; THC, total hospital costs; LOS, length of stay; US, United States; MI, myocardial infarction; AV, atrioventricular				

Impact of PCI versus CABG on secondary outcomes in the propensity score-matched cohort

In the propensity score-matched analysis, secondary outcomes were evaluated between the two matched cohorts. The overall mean LOS was 9.8 days (SD 3.2), and the mean hospital cost was $US 225,156 (SD, $15,438). Notable differences between the CABG and PCI cohorts were identified in the incidence of acute myocardial infarction (4.3% vs. 2.7%; P<0.001), acute ischemic stroke (1.7% vs. 1.2%; P=0.008), complete atrioventricular block (1.6% vs. 1.2%; P=0.035), sepsis (3.6% vs. 1.8%; P<0.001), atrial flutter (17.9% vs. 6.9%; P<0.001), atrial fibrillation (33% vs. 15%; P<0.0001), and ventricular fibrillation (2.1% vs. 1.4%; P=0.046). Furthermore, CABG was associated with a longer hospitalization duration (15% vs. 5.9%; P<0.001) and higher mean hospital costs ($US 320,917 vs. $129,396; P<0.001). The incidence of acute kidney injury was greater in the CABG cohort (31.9% vs. 29.5%; P=0.011). However, the requirement for postoperative dialysis was more prevalent in the PCI cohort (9.9% vs. 7.5%; P<0.001).

## Discussion

We examined a cohort of 151,270 patients hospitalized for multivessel CTO, who underwent either PCI or CABG. The findings indicate that patients undergoing CABG experience higher mortality rates, longer hospital stays, and increased hospital costs compared to those receiving PCI. Specifically, the CABG cohort exhibited a 5.8% mortality rate, in contrast to 3.4% in the PCI group. This elevated mortality rate among CABG patients is consistent with existing literature, which underscores the complexity and invasiveness of CABG, particularly in patients with additional comorbidities [34]. Previous research has reported similar 30-day mortality rates for patients undergoing CABG or PCI [35]. The higher in-hospital mortality associated with the invasiveness of CABG is counterbalanced by its effective and lasting revascularization, potentially aligning the 30-day mortality rates with those of patients undergoing PCI. Conversely, the initial lower risk associated with PCI may be offset over time by subsequent complications or disease progression, equalizing its 30-day mortality rate with that of CABG. These outcomes are further influenced by comorbidities and the nuances of posttreatment care. Additionally, the interpretation of mortality rates is affected by methodological research approaches, such as the adjustment of confounding variables. Thus, the varied mortality rates observed following these interventions reflect a complex interplay of procedure type, patient health, and challenges in managing chronic conditions, such as chronic kidney disease (CKD) and cardiovascular diseases. A recent study reported higher rates of one-year all-cause mortality, major adverse cardiac and cerebrovascular events, and subsequent revascularization in patients with CAD treated with PCI compared to those undergoing CABG [36].

The extended duration of hospitalization and the increased financial burden observed in the CABG group, with mean hospital costs being significantly higher for CABG compared to PCI, reflect the more invasive nature of CABG. However, recent evidence indicates that the direct costs of PCI and CABG may be comparable, and that other factors, such as costs associated with travel or loss of labor productivity, may influence the overall cost differences between the two approaches [37]. According to patient perspectives measured by the Seattle Angina Questionnaire (SAQ) and Short Form of 36 items (SF-36) instruments, CABG demonstrated cost-effectiveness. There was a reduction of $16,581 and $34,543 for every increase in effectiveness as per the SAQ and the SF-36 evaluations, respectively [38]. While patients undergoing CABG for MVD CTO can anticipate longer initial hospitalizations and higher average costs, evidence suggests that CABG may be more cost-effective in the long term. Further prospective studies in this cohort are necessary to confirm this finding.

Our study further elucidates various predictors of CABG, including ethnicity, insurance type, and specific comorbidities such as dyslipidemia and obesity. The preference for CABG over PCI in certain demographic groups or based on insurance type raises significant questions regarding treatment accessibility and decision-making within this patient population. Our findings underscore the impact of demographic and clinical factors in determining the treatment pathway for patients with CTO and MVD. Additionally, the current findings reveal that the CABG cohort experienced a higher incidence of acute complications, including myocardial infarction, stroke, and various cardiac arrhythmias. According to the Society of Thoracic Surgeons CABG Adult Cardiac Surgery Database (STS ACSD), the incidence of ischemic stroke post-CABG decreased from 1.6% to 1.2% between 2000 and 2009 [39]. However, our findings suggest that this rate may be elevated in patients with MVD. The occurrence of stroke following CABG or PCI is associated with a fourfold increase in all-cause mortality over a five-year period, extending beyond the immediate periprocedural phase [40].

The index study demonstrated that the cohort undergoing CABG exhibited a higher incidence of adverse cardiovascular events, including atrioventricular blocks and both atrial and ventricular arrhythmias. In contrast, the PCI cohort experienced elevated rates of acute myocardial infarction. This finding suggests a greater potential for acute cardiac complications in patients undergoing CABG. Nevertheless, a recent meta-analysis involving 1,198 patients indicated that CABG was associated with a reduced risk of long-term major adverse cardiac and cerebrovascular events, cardiac mortality, and the necessity for repeated long-term revascularization and cerebrovascular accidents compared to PCI. The long-term recurrence rates of myocardial infarction were found to be similar between the CABG and PCI groups [41,42]. Acute kidney injury (AKI) is commonly observed following multivessel coronary revascularization, with a higher incidence noted after CABG compared to PCI. Previous research has indicated that CABG is associated with a two- to three-fold increase in the adjusted odds of developing AKI relative to PCI [43,44]. Consistent with these prior findings, the index study demonstrated an elevated risk associated with CABG and suggested a potentially more severe form of AKI in the PCI cohort, as evidenced by a greater number of patients requiring postoperative dialysis in this group.

Clinical implications

The observation that PCI was associated with lower in-hospital mortality, shorter hospital stays, and reduced costs suggests that PCI may be a more favorable revascularization strategy for select patients with CTO and multivessel disease, particularly those at high surgical risk, those with significant comorbidities and less complicated coronary anatomy. Conversely, the lower rates of recurrent myocardial infarction observed with CABG highlight its potential role in achieving more durable revascularization in appropriately selected patients such as patients with prior PCI or complex coronary anatomy. These results underscore the importance of individualized treatment planning that integrates patient comorbidities, anatomical complexity, procedural risk, and long-term goals of care. Multidisciplinary cardiovascular teams may utilize these findings to refine patient selection and counseling, ensuring that revascularization strategies are aligned with both clinical outcomes, goals of care, and resource utilization. Ultimately, tailoring therapy based on risk stratification and shared decision-making has the potential to optimize both short-term safety and long-term benefits for patients with CTO and multivessel disease.

The index findings, by emphasizing the short-term benefits of PCI derived from real-world data in the United States, provide valuable insights for clinicians who may utilize "staged" or "interval" PCI as a provisional measure while optimizing comorbidities and planning CABG. Although not formally classified in the current guidelines, this approach is supported in several respects. Firstly, guidelines advocate for staged PCI in selected patients with acute coronary syndrome and multivessel disease, such as performing culprit-first PCI followed by non-culprit revascularization, thereby reflecting the acceptability of stepwise strategies when immediate full revascularization is not ideal [45]. Secondly, hybrid coronary revascularization explicitly combines CABG and PCI in either sequence (PCI→CABG or CABG→PCI) based on Heart-Team judgment, which essentially constitutes a structured "interval" strategy when anatomical considerations and risks favor distributing the procedures over time [46]. Thirdly, when PCI precedes CABG, perioperative antiplatelet bridging, such as short-acting intravenous cangrelor, is discussed in consensus reviews to safely pause P2Y12 therapy before surgery, underscoring that this sequence is anticipated in practice [47]. In summary, employing PCI as a bridge to definitive CABG is an accepted, Heart-Team-driven strategy for stabilization or symptom relief when immediate surgery is not optimal.

In the management of CTO with multivessel disease, clinical decision-making is influenced by a combination of anatomical, procedural, patient, and system-level determinants. Factors such as ethnicity, socioeconomic status, and insurance coverage can impact access to advanced revascularization techniques, potentially resulting in biased treatment selection. Black and Hispanic patients are consistently less likely to receive advanced revascularization procedures compared to White patients, even when controlling for insurance status and comorbidities [48]. Underserved populations may be more likely to receive PCI rather than CABG due to perceived procedural risks or financial constraints [49,50]. Furthermore, comorbidities, including diabetes, chronic kidney disease, or advanced frailty, often lead physicians to favor less invasive methods, even when CABG might offer superior long-term outcomes [51]. Hospital practices, such as the availability of hybrid operating rooms, surgical expertise, and interventional cardiology resources, further contribute to variability in treatment strategies [52]. Physician preferences and implicit biases may also influence decision-making, with some clinicians opting for the modality with which they are more familiar or comfortable, rather than relying solely on objective risk-benefit analyses [53]. These factors highlight the necessity for a multidisciplinary, guideline-based, and equity-focused approach to ensure that patients with CTO and multivessel disease receive consistent, evidence-based, and patient-centered care.

Strengths and limitations

The strengths of the current study include its large cohort size and the application of propensity score matching. However, this study has some limitations. As a retrospective analysis of administrative data from the NIS, it is susceptible to coding errors and residual confounding, even with the application of propensity score matching. Nevertheless, given that ICD-10-CM/PCS codes are derived from discharge abstracts that undergo rigorous review and are crucial for billing and reimbursement, the probability that PCI or CABG procedures were systematically omitted, misclassified, or miscoded at a frequency sufficient to significantly alter the outcomes in this extensive nationwide cohort is considered negligible. The NIS database lacks clinical details on coronary anatomy, lesion complexity, procedural techniques, operator experience, and in-hospital medication use, which limits our ability to comprehensively account for all factors influencing treatment choice and outcomes. CTO was defined using ICD-10 code I29.82, which does not specify the number of coronary arteries occluded. Furthermore, the study exclusively evaluated acute in-hospital outcomes, precluding the assessment of long-term mortality, recurrent ischemic events, and quality-of-life measures. Due to the anonymization of each hospitalization in the database, tracking the readmission of specific individuals is not feasible. To address this limitation, all hospitalizations involving a prior PCI or CABG were excluded from the study cohort (see Appendix 1 for ICD-10 codes for old PCI and old CABG). While the large, national sample enhances the generalizability of the findings, and the application of propensity score matching reduces significant bias, it remains impossible to draw causal inferences regarding the comparative effectiveness of PCI versus CABG in CTO with multivessel disease from this observational design. Furthermore, residual imbalance cannot be entirely eliminated without the implementation of randomization. Prospective, randomized studies are thus necessary to further validate these findings and inform clinical decision-making in this complex patient population. Given that the study cohort included white Americans, Blacks, Hispanics, Asians and Pacific Islanders, and other mixed races, the findings are easily generalizable to similar mixed populations.

The results of this study have significant implications for clinical decision-making in the management of patients with multivessel chronic total occlusions. Clinicians should continue to employ an individualized approach that takes into account the unique risk profile of each patient.

## Conclusions

In this retrospective observational study, PCI was associated with lower in-hospital mortality, shorter lengths of hospital stay, and significantly lower hospitalization costs compared with CABG. This disparity in mortality may be attributed to residual confounding, particularly due to unmeasured variables related to procedural risk. Conversely, hospitalizations involving CABG were associated with lower incidence of recurrent acute myocardial infarction. However, this benefit was offset by higher rates of acute postprocedural complications, including arrhythmias, stroke, and sepsis. The index study thus generates the hypotheses that PCI may offer distinct advantages in the acute hospital setting, while CABG may confer benefits in reducing recurrent ischemic events. Additional prospective randomized studies that sufficiently integrate patient-, provider-, and institutional-level factors as well as left ventricle function, lesion complexity, and comorbidities are necessary to validate these findings and inform broad practice changes in the real-world setting.

## Appendices

Appendix 1 

**Table 5:** ICD-10-CM and ICD-10-PCS Codes for Study Cohort Identification This table lists the ICD-10-CM and ICD-10-PCS codes used to identify chronic total occlusion, multivessel coronary artery disease, percutaneous coronary intervention, coronary artery bypass grafting procedures, and complications in the Nationwide Inpatient Sample (NIS) database. Codes were compiled from the Centers for Medicare & Medicaid Services. (2025). ICD-10-CM/PCS. CMS. https://www.cms.gov/medicare/icd-10/2025-icd-10-cm, and were validated against prior studies and refined for accuracy to ensure alignment with standard NIS-based research methodologies. ICD-10-CM, International classification of disease, tenth edition, clinical modification; ICD-10-CM, International classification of diseases, tenth edition, procedure coding system; CRRT, continuous renal replacement therapy; PCI, percutaneous coronary intervention; CABG, coronary artery bypass graft

ICD-10 Code	Description
I25.82	Chronic total occlusion of coronary artery
I25.1x and I25.7xx	Multivessel coronary disease
I25.10	Atherosclerotic heart disease of native coronary artery without angina pectoris
I25.11	Atherosclerotic heart disease of native coronary artery with angina pectoris
I25.118	Atherosclerotic heart disease of native coronary artery with other forms of angina pectoris
I25.119	Atherosclerotic heart disease of native coronary artery with unspecified angina pectoris
I25.700–I25.799	Atherosclerosis of coronary artery bypass graft(s) with or without angina pectoris (single/multiple/unspecified vessels)
02703ZZ	Dilation of one coronary artery with drug-eluting intraluminal device, percutaneous approach
02713ZZ	Dilation of two coronary arteries with drug-eluting intraluminal device, percutaneous approach
02723ZZ	Dilation of three coronary arteries with drug-eluting intraluminal device, percutaneous approach
02733ZZ	Dilation of four or more coronary arteries with drug-eluting intraluminal device, percutaneous approach
02100Z9	Bypass coronary artery, one site from aorta with autologous venous tissue, open approach
021109W	Bypass coronary artery, one site from aorta with synthetic substitute, open approach
021209W	Bypass coronary artery, two sites from aorta with synthetic substitute, open approach
02100A9–021339W	Family of CABG codes: Bypass coronary artery (1–4+ sites) from aorta with venous/arterial/synthetic grafts, open approach
N17.0	Acute kidney failure with tubular necrosis
N17.1	Acute kidney failure with acute cortical necrosis
N17.2	Acute kidney failure with medullary necrosis
N17.8	Other acute kidney failure
N17.9	Acute kidney failure, unspecified
I21.0	ST elevation (STEMI) myocardial infarction of anterior wall
I21.1	ST elevation (STEMI) myocardial infarction of inferior wall
I21.2	ST elevation (STEMI) myocardial infarction of other sites
I21.3	ST elevation (STEMI) myocardial infarction of unspecified site
I21.4	Non-ST elevation (NSTEMI) myocardial infarction
I21.9	Acute myocardial infarction, unspecified
I63.9	Cerebral infarction, unspecified
I63.0–I63.8	Cerebral infarction due to thrombosis, embolism, or occlusion of specific arteries
I61.9	Nontraumatic intracerebral hemorrhage, unspecified
I61.0–I61.8	Nontraumatic intracerebral hemorrhage, specified sites
I44.0	Atrioventricular block, first degree
I44.1	Atrioventricular block, second degree
I44.2	Atrioventricular block, complete (third degree)
I45.9	Conduction disorder, unspecified
A41.9	Sepsis, unspecified organism
A41.0–A41.8	Sepsis due to specific organisms (e.g., staphylococcus, streptococcus, E. coli, other gram-negative organisms)
R65.20	Severe sepsis without septic shock
R65.21	Severe sepsis with septic shock
I48.0	Paroxysmal atrial fibrillation
I48.1	Persistent atrial fibrillation
I48.91	Unspecified atrial fibrillation
I48.3	Typical atrial flutter
I48.4	Atypical atrial flutter
I48.92	Unspecified atrial flutter
I49.01	Ventricular fibrillation
I49.02	Ventricular flutter
5A1D00Z	Performance of urinary filtration, intermittent, external approach (hemodialysis)
5A1D60Z	Performance of urinary filtration, continuous, external approach (continuous hemodialysis/CRRT)
Z95.5	Presence of coronary angioplasty implant and graft (Old PCI)
Z95.1	Presence of aortocoronary bypass graft (Old CABG)

Appendix 2 

**Table 6:** Missing covariate data n = frequency; % = proportion

Variable	n	%
Unmatched cohort		
Age	2	0
Admission month	52	0.05
Died	68	0.07
Discharge status	68	0.07
Discharge quarter	52	0.05
Elective vs. non-elective admission	164	0.16
Female	18	0.02
Primary payer/ Insuranc status	85	0.09
Patient location	263	0.26
Race	2,894	2.91
Total hospital charges	1,339	1.35
Median income quartile in the patient’s ZIP code	1,559	1.57
Matched cohort		
In-hospital mortality	2	0.05
Elective vs nonelective admission	9	0.24

Appendix 3 

**Table 7:** Evaluation of Study Design, Interpretation, and Analysis Considerations Adapted from methodological checklist for studies published using the nationwide inpatient sample database, Khera et al. 2017 [21]

Criterion	Addressed in this Study?	Commentary
Section A: Study Framework		
Does the analysis recognize the inherent limitation that only conditions and procedures identifiable during hospitalization can be captured?	✔️ Yes	The study acknowledges that only inpatient data are observable, given the hospital-based nature of the dataset.
Is it clear that the unit of analysis is hospital encounters rather than individual patients?	✔️ Yes	The manuscript consistently refers to "hospitalizations" instead of "patients" to reflect this distinction.
Section B: Clinical Coding and Outcome Scope		
Are the diagnostic and procedural codes used in the study based on prior validation or literature?	✔️ Yes	All codes used to define conditions and procedures were sourced from validated studies and/or previously published coding frameworks.
Are outcomes restricted to the in-hospital period?	✔️ Yes	Only events occurring during the index hospitalization were evaluated.
Are complications and comorbidities clearly differentiated where possible?	✔️ Yes	The study separates complications from pre-existing conditions unless the data structure makes this distinction infeasible.
Section C: Analytical Approach		
Does the analysis account for the complex survey design of the NIS?	✔️ Yes	The analyses appropriately incorporate the NIS's sampling framework— including clustering (HOSP_NIS), weighting (DISCWT), and stratification (NIS_STRATUM)—using SAS procedures (e.g., SURVEYLOGISTIC, SURVEYFREQ).
Are temporal changes in the NIS data structure properly addressed in longitudinal analyses?	✔️ Yes	Modifications over time in database structure and coding have been explicitly handled in the methodology section.

Appendix 4 

**Table 8:** Balance Statistics After Propensity Score Matching Between PCI and CABG Groups PCI, percutaneous coronary intervention; CABG, coronary artery bypass grafting; χ², Chisquare statistic; HMO, health maintenance organization; APR-DRG, all patient refined-diagnosis related groups;

Variable	PCI (N=9,210)	CABG (N=9,210)	P-value	χ²	Standard Error	Standardized Mean Difference	Interpretation
Median age (years)	66.0	65.9	0.897	5.00	0.08	0.01	Balanced
Sex (Male, %)	61.3	61.3	0.999	0.00	0.07	0.00	Perfect balance
Insurance type			0.852	0.309	0.04	0.01	Balanced
Medicare	60.0	59.6					
Medicaid	12.3	12.2					
Private/HMO	23.7	23.8					
Self-pay	4.1	4.4					
Race			0.979	0.02	0.01	0.00	Balanced
White	50.2	50.6					
Black	21.2	20.7					
Hispanic	14.8	14.5					
Asian/Pacific Islander	6.2	6.2					
Native American	1.8	1.3					
Other	5.8	5.7					
Nonelective admissions (%)	90.5	90.0	0.981	0.02	0.006	0.01	Balanced
Weekend admissions (%)	68.9	67.3	0.880	0.03	0.08	0.02	Balanced
APR-DRG Severity of Illness			0.942	0.04	0.03	0.02	Balanced
Minor	4.7	4.5					
Moderate	34.0	32.9					
Major	42.3	41.8					
Extreme	19.0	20.8					
APR-DRG Risk of Mortality			0.868	0.05	0.07	0.02	Balanced
Minor	6.5	6.5					
Moderate	36.4	36.4					
Major	37.7	33.6					
Extreme	19.3	23.5					
Hospital region			>0.999	0.02	0.01	0.00	Balanced
Northeast	13.1	13.6					
Midwest	20.1	19.1					
South	44.6	44.0					
West	22.2	23.3					
Urban teaching hospital (%)	69.8	67.9	0.878	0.07	0.02	0.02	Balanced
Hospital ownership			0.910	0.03	0.10	0.03	Balanced
Government	9.2	7.6					
Private nonprofit	73.7	74.7					
Investor-owned	18.1	17.7					
Comorbidities							
Obesity	24.0	24.1	0.868	0.03	0.01	0.00	Balanced
Hypertension	1.6	1.8	>0.999	0.01	0.03	0.01	Balanced
Dyslipidemia	65.2	65.4	0.882	0.03	0.05	0.01	Balanced
Tobacco use	43.8	42.3	0.886	0.03	0.002	0.02	Balanced
Peripheral vascular disease	17.7	16.8	>0.999	0.02	0.04	0.02	Balanced
Congestive cardiac failure	57.8	56.7	>0.999	0.02	0.03	0.02	Balanced
Previous stroke	8.8	14.4	0.375	0.03	0.08	0.06	Balanced
COPD	24.3	26.3	0.870	0.03	0.009	0.02	Balanced
Dementia	3.0	1.3	0.757	0.04	0.04	0.02	Balanced
Diabetes (Types 1 & 2)	66.2	65.2	>0.999	0.02	0.10	0.01	Balanced
Chronic liver disease	3.2	3.5	>0.999	0.02	0.01	0.01	Balanced
Malignant neoplasia	2.9	2.4	>0.999	0.02	0.03	0.02	Balanced
Rheumatoid disease	3.0	2.6	0.746	0.04	0.09	0.02	Balanced
Hemiplegia	1.0	1.2	0.745	0.04	0.04	0.01	Balanced
